# Enhancing the Efficacy of CAR-T Cell Therapy: A Comprehensive Exploration of Cellular Strategies and Molecular Dynamics

**DOI:** 10.33696/cancerimmunol.6.080

**Published:** 2024

**Authors:** Mehmet A. Baysal, Abhijit Chakraborty, Apostolia M. Tsimberidou

**Affiliations:** 1 Department of Investigational Cancer Therapeutics, The University of Texas MD Anderson Cancer Center, Houston, TX, USA

**Keywords:** CAR-T cell therapy, T cell subsets, CD4^+^ T cells, Innate-like T lymphocytes, Cord blood-derived hematopoietic stem cells, Interleukin-15 (IL-15), Tumor targeting, Cancer immunotherapy

## Abstract

The emergence of chimeric antigen receptor T cell (CAR-T cell) therapy has revolutionized cancer treatment, particularly for hematologic malignancies. This commentary discusses developments in CAR-T cell therapy, focusing on the molecular mechanisms governing T cell fate and differentiation. Transcriptional and epigenetic factors play a pivotal role in determining the specificity, effectiveness, and durability of CAR-T cell therapy. Understanding these mechanisms is crucial to improve the efficacy and decrease the adverse events associated with CAR-T cell therapies, unlocking the full potential of these approaches. T cell differentiation in CAR-T cell product manufacturing plays an important role in clinical outcomes. A positive correlation exists between the clinical efficacy of CAR-T cell therapy and signatures of memory, whereas a negative correlation has been observed with signatures of effector function or exhaustion. The effectiveness of CAR-T cell products is likely influenced by T-cell frequency and by their ability to proliferate, which is closely linked to early T cell differentiation. The differentiation process involving distinct T memory cell subsets is initiated upon antigen elimination, indicating infection resolution. In chronic infections or cancer, T cells may undergo exhaustion, marked by continuous inhibitory receptor expression, decreased cytokine production, and diminished proliferative capacity. Other cell subsets, such as CD4^+^ T cells, innate-like T lymphocytes, NKT cells, and cord blood-derived hematopoietic stem cells, offer unique advantages in developing the next-generation CAR-T cell-based therapies. Future research should focus on optimizing T-cell-enhancing approaches and developing strategies to potentially cure patients with hematological diseases and solid tumors.

## Introduction

The emergence of chimeric antigen receptor T cell (CAR-T cell) therapy has enriched the therapeutic strategies for cancer, particularly hematologic malignancies. This approach involves genetically modifying T cells to express a CAR that can identify and target cancer cells. CAR-T cell therapies have been successful in treating B-cell non-Hodgkin lymphoma, follicular lymphoma, B cell acute lymphoblastic leukemia, and multiple myeloma, and these successes have led to global regulatory approvals.

In our recent article, we explored the latest developments in CAR-T cell therapy, focusing on the molecular mechanisms that determine T cell fate and differentiation [1]. Understanding these mechanisms is crucial to unlocking the full potential of CAR-T cell therapies. Transcriptional and epigenetic factors play an important role in determining the specificity, effectiveness, and durability of CAR-T cell therapy. The challenges posed by different T cell subsets and the complexities associated with targeting various antigens indicate the need to develop next-generation CAR-based therapeutic modalities [1,2]. The aim of this commentary is to provide a comprehensive description of the molecular dynamics governing T cell fate and differentiation within the context of CAR-T cell therapy. The intricate roles of T cell subsets, including CD4^+^ T cells, innate-like T lymphocytes such as γδ T cells and natural killer T (NKT) cells, and cord blood-derived hematopoietic stem cells, are described. The primary focus is to elucidate the potential roles of these T cell subsets in the advancement of next-generation CAR-T therapies.

## T cell Differentiation in CAR-T cell Manufacturing and Clinical Outcomes

Recent research has focused on the significance of T cell differentiation in the manufacturing of CAR-T cell products and its influence on clinical outcomes [3–9]. Utilizing the transcriptomic signatures based on the differential expression of memory-, effector-, and dysfunction-associated genes has enabled the characterization of cell therapies and the correlation of product characteristics with clinical response [3–9]. Studies have demonstrated a positive correlation between the clinical efficacy of CAR-T cell therapy and signatures of memory, while a negative correlation has been observed with signatures of effector function or exhaustion [10,11]. Despite these findings and significant advancements in CAR-T cell therapy, there is still a lack of clear understanding about the specific attributes of the therapeutic product that lead to a favorable response.

Recently, methods have been employed to enhance the stemness and potency of CAR-T cell products, including shortening the manufacturing process and stimulating T cells to express key molecules [12]. For example, the use of the inflammatory cytokine interleukin 12 (IL-12) has been found to induce high levels of CD62L expression, and it skews T cells towards terminal effector differentiation [13]. Additionally, *in vitro* studies have demonstrated that T cells can be stimulated to re-express CD62L and CCR7 by T-cell receptor (TCR), interleukin-2 (IL-2), or interleukin-21 (IL-21) [14]. Moreover, the tumor necrosis factor receptor family member CD27 promotes memory formation by maintaining the expression of interleukin-7 receptor-α (IL-7RA) independent of IL-2 [15,16]. Research has indicated that the co-expression of CD27 with CD45RO [8] or CCR7 [7] correlates with efficacy, suggesting that CD27 may serve as a better predictive tool than the homing markers CD62L and CCR7, when considered in isolation.

The effectiveness of CAR-T cell products is likely influenced not just by the number of T-cells but also by their ability to proliferate, which is closely linked to the early differentiation of T cells. Because the composition of CAR-T cell products is critical for clinical success, it is important to understand the T cell differentiation process comprehensively.

## Dynamic Transitions in T cell Phenotypes

In the expansion phase of the immune response, effector T cells (Teff) play a vital role in clearing antigens. Subsequently, Teff cells transition to long-lasting memory T cells (Tmem) during the contraction phase [17]. Tmem cells possess greater developmental and proliferative potential than Teff cells [17]. They can recruit additional capabilities, such as high spare respiratory capacity. Tmem cells have the unique characteristic of homing to secondary lymphoid organs or circulating in the periphery, identified by increased expression of IL-7RA, that enables them to undergo homeostatic proliferation even in the absence of TCR stimulation. Moreover, Tmem cells exhibit levels of homing molecules, depending on the organ where they are localized, including CD62L and CCR7 in lymphoid organs, CX3CR1 in the periphery, and CD103 and Hobit in tissues [18]. As T cells differentiate, they start to display effector function characteristics, such as enhanced cytotoxic activity and cytokine production. However, they gradually lose their ability to proliferate.

## T cell Differentiation and Exhaustion in Persistent Infections and Cancer

T cell exhaustion, in which T cells lose their effector functions and proliferative capacity, is typically observed in chronic infections and cancer [10]. In chronic infections, pathogens are not eliminated rapidly but persist and result in chronic antigen stimulation and persistent inflammation, potentially leading to exhaustion and/or clonal deletion of pathogen-specific CD8 T cells [19–23]. T cell exhaustion has been associated with chronic viral, bacterial, and parasitic infections [19], and it has been extensively studied in the context of persistent viral infections, such as human immunodeficiency virus (HIV) and hepatitis B virus (HBV) [19–21]. Additionally, T cell exhaustion has been investigated in the setting of viral infections [23], chronic inflammatory diseases [22] and various tumor types [24]. The differentiation process of T cells and the development of exhaustion are influenced by various factors, including the expression of inhibitory receptors (e.g., PD-1, LAG3), cytokine production, and the activity of specific transcription factors (e.g., TCF-1, TOX, FOXO1) [18,25]. Cancer is linked to T-cell exhaustion across tumor types. In addition, in patients with glioma, this exhaustion contributes to immunotherapy failure [24].

The differentiation of T cells, which involves the generation of distinct Tmem cell subsets, is initiated upon the elimination of the antigen, indicating the resolution of the infection. In chronic infections or cancer, T cells follow a different path of development called T cell exhaustion. This state is marked by continuous expression of inhibitory receptors or checkpoint inhibitors like programmed cell death protein 1 (PD-1), lymphocyte activation gene 3 (LAG3), 2B4, and cytotoxic T-lymphocyte-associated antigen 4 (CTLA-4); decreased production of effector cytokines such as IFN-γ and tumor necrosis factor (TNF); and diminished proliferative capacity [10].

**Figure 1.**
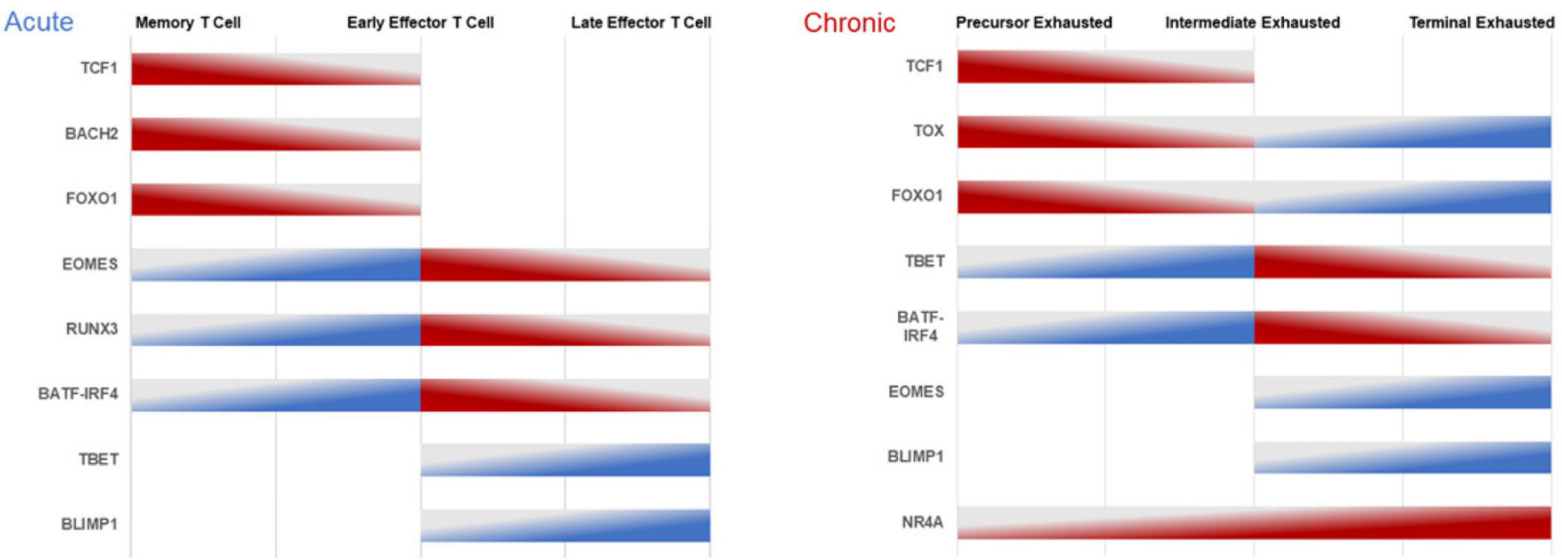
Expression levels of key regulators of T-cell differentiation and exhaustion in acute and chronic conditions. **Abbreviations:** BACH2: Broad complex-tramtrack-bric a brac and Cap’n’collar homology 2; BATF: B cell activating transcription factor; BLIMP-1: B lymphocyte-induced maturation protein 1; EOMES: Eomesodermin; FOXO1: Forkhead O transcription factors-1; IRF4: Interferon regulatory factor 4; NR4A: Nuclear receptor-4A; RUNX3: Runt-domain transcription factors-3; TBET: T-box expressed in T cells; TCF-1: T cell factor-1; TOX: Thymocyte selection-associated high-mobility group box

Transcription factors are proteins that regulate gene expression by binding to specific DNA sequences in the genome. Transcription factors such as thymocyte selection-associated high mobility group box (TOX), B-cell-activating transcription factor (BATF), interferon regulatory factor 4 (IRF4), and nuclear factor of activated T cell (NFAT) play critical roles in regulating T cell exhaustion (Figure 1) [19]. Transcription factors also influence various aspects of the immune system and form a complex regulatory network that organizes the immune system’s response to various challenges and threats. This network leads to poor results of CAR-T cell therapy in the treatment of solid tumors [25].

Other transcription factors include BACH2, which is an essential transcription factor for B-cell development and function, impacting the immune response [26]. BATF contributes significantly to T cell differentiation and function, influencing immune responses [27]. B-lymphocyte-induced maturation protein 1 (BLIMP-1) is a transcriptional repressor crucial for differentiating plasma cells and is responsible for antibody production [28]. Eomesodermin (EOMES) is vital for the development and function of CD8^+^ T cells and NK cells in immune defense [29]. Forkhead box protein (FOXO1) regulates T cell development and function within the immune system [30]. IRF4 plays a critical role in B and T helper cell differentiation. Nuclear receptor 4A (NR4A) regulates immune cell development and function [31]. Runt-related transcription factor 3 (RUNX3) is indispensable for the development and function of CD8+ T cells and regulatory T cells, contributing to immune responses [32]. TBET, critical for T helper 1 (Th1) cell differentiation, is essential for cellular immune responses against infections [33]. T-cell factor 1 (TCF-1) is involved in developing and maintaining T cells and regulating T cell receptor signaling [34]. TOX is involved in developing and maintaining T cells, particularly in the context of T cell exhaustion during chronic infections and cancer [35].

The differentiation process that leads to terminal exhaustion is similar to the differentiation of effector T cells that occurs with acute stimulation (Figure 1). Tpex cells (precursor exhausted T-cells), a subtype of dysfunctional T cells, were identified in a chronic viral infection model [36]. These Tpex cells express TCF-1 (T cell factor-1) and can self-renew and differentiate into Tex (effector-like exhausted T cells) that have similarities with peripheral Tmem cells. Elevated levels of TCF-1+ Tpex cells in human cancer have been associated with longer overall survival and improved treatment outcomes. This indicates that Tpex cells play a critical role in sustaining T cell responses over time [37–40]. However, the persistent presence of antigens eventually gives rise to terminally differentiated and exhausted T cells (Tex^term^).

In acute and chronic conditions, the expression levels of key regulators play a vital role in T cell differentiation and exhaustion (Figure 1). Tmem cells exhibit the highest TCF-1 [34], BACH2 [26], and FOXO1 [30] expression during acute TCR stimulation; these regulators gradually decrease as the cells undergo effector differentiation. Conversely, EOMES [29], RUNX3 [32], and BATF-IRF4 [31, 41] expression is initially low in Tmem cells, peaks in early Teff cells, and then declines in late Teff cells. Furthermore, the gradual increase in the expression of T-BET [33] and BLIMP-1 [42] contributes to effector T-cell differentiation. Tpex cells demonstrate the highest TCF-1 [27,43], TOX [35,43], and FOXO1 [27,44,45] expression during chronic TCR stimulation, gradually decreasing as they become exhausted. As T cells become terminally exhausted, the expression of TOX and FOXO1 gradually increase, while the expression of TCF-1 remains low. However, during chronic TCR engagement, the expression of T-BET [33,43,46], BATF-IRF4 [27,47], and NR4A [48] is the lowest in Tpex cells. The expression of T-BET and BATF-IRF4 peaks in intermediate exhausted T cells and then decreases, while the expression of NR4A remains elevated and reaches its peak at the Tex^term^ cell stage. Moreover, the expression of EOMES [43,46,49] and BLIMP-1 [28] begins later at the intermediate exhausted stage, increases gradually, and peaks at the terminal exhausted stage.

### Modulators of acute and chronic T cell differentiation

The differentiation of T cells under both acute and chronic stimulation is a gradual process closely regulated by epigenetic and transcriptional modulators. The proper commitment to T cell fate relies on the precise abundance and timing of expression of these modulators. Notably, while chronic and acute stimulation lead to quite different outcomes, there is considerable overlap in the types of modulators involved in each process, as described below.

### Differential gene expression control during acute antigenic challenge

During the initial phases of an infection, most T cells undergo differentiation into short-lived effector cells (SLECs). In contrast, a specific subgroup of activated T cells known as memory precursor effector cells (MPECs) are primed to transition into enduring, self-renewing memory T cells. This strategic differentiation is crucial to safeguarding the host against future infections [50–52]. Collectively, the regulated expression of conflicting transcription factors controls the differentiation of CD8^+^ T cells and determines their fate, leading toward either memory or effector T cell functionality.

### Differential gene expression control during chronic antigenic challenge

In chronic antigen stimulation, T cell exhaustion occurs irrespective of inflammatory signals and environmental parameters. The extent of exhaustion correlates directly with the antigen’s quantity and the duration/frequency of TCR stimulation, independently of other factors [47,53]. It is important to highlight that, separate from T cell exhaustion, the tumor microenvironment can induce T cell dysfunction and T-cell function can be impaired by tumor-associated hypoxia, acidity, and altered lipid metabolism, factors that may restrict CAR-T function. The functionality of CAR-T cells can also be hindered by immunosuppressive factors in the microenvironment [28,48,49,54].

## Modulating CAR-T cell Differentiation via Cellular Engineering

Despite the clinical advantages associated with a substantial memory composition in CAR-T cell therapies, the enhancement of memory function has not been proven in CAR-T cells developed under conditions favoring memory differentiation. The association between markers associated with memory (CD62L or CCR7) in CAR-T cells and response is study-dependent and may not be a suitable indicator of CAR-T cell efficacy; CD27 might be a better indicator. Attempts to bolster memory in the final product have not resulted in improved clinical outcomes. Therefore, this section will focus on engineering strategies to improve the stemness and potency of CAR-T cell products by considerably reprograming CAR-T cell differentiation and exhaustion states. Recent reviews have explored the broader scope of next-generation CAR-T enhancements, going beyond the manipulation of differentiation and exhaustion states [55,56]. Factors associated with T cell memory or dysfunction, assessed within the framework of adoptive cell therapy, are BCL6, cJUN, TCF-1, FOXO1, BATF, and others [1], and their modification can enhance memory function or prevent dysfunction and exhaustion.

An emerging approach that shows promise in preclinical models involves inhibiting factors responsible for T cell exhaustion. Deletion of DNMT3A (DNA methyltransferase 3A) can preserve the antitumor activity of CAR-T cells during prolonged tumor exposure [57]. Furthermore, in a model of bacterial infection, depletion of SUV39H1 – a histone lysine methyltransferase that is involved in stem/memory gene silencing—increased long-term memory and survival [58]. Other studies have focused on the NR4A family of orphan nuclear receptors and the TOX family of DNA-binding proteins (high-mobility group box), which are downstream targets of NFAT. Studies have demonstrated antitumor responses in CD8+ tumor-infiltrating T lymphocytes by deleting NR4A1 [59] and all three NR4A family members [60] to reinstate AP-1 function. In human CAR-T cells, functional ablation of PD1 increased the antitumor activity across diverse xenograft models of solid tumors [61–63]. The elimination of PD1 initially increases the proliferation of antiviral T cells in the expansion phase of chronic viral infection [64]. Nevertheless, in the subsequent contraction phase, as PD1 is eliminated, the natural decline of antiviral T cells is accelerated, ultimately resulting in a diminished presence of memory cells [64,65]. These reports reveal that dysfunctional T cells in cell therapy can benefit from PD1 deletion. Alternatively, in the context of chronic infections, the upregulation of PD1 may support the prolonged persistence of effector cells, contributing to long-lasting immune responses [66], e.g., high tumor burden may result in prolonged antigen exposure and a more prominent state of exhaustion in the cell therapy product [67].

Therefore, CAR-T cells are likely to be more effective if used earlier in the course of the disease in patients with solid tumors or hematologic malignancies. Theoretically, CAR-T cells should be used as maintenance therapy for patients with high-risk disease that have responded to initial treatment and have a low tumor volume.

## Ongoing Strategies to Optimize T-cell Therapy

Other cell subsets, such as CD4+ T cells, innate-like T lymphocytes, and cord blood-derived hematopoietic stem cells, offer unique advantages in developing next-generation CAR-T based therapies.

### CD4^+^ T cells

Further research is needed to advance the understanding of the potential of CD4^+^ T cells in CAR-T therapy. In preclinical tumor models, it was shown that CD4^+^ T cells with an under-differentiated phenotype can significantly improve the therapeutic activity of T cells [68,69]. Besides their helper function, CD4^+^ CAR-T cells can induce potent cytotoxic responses and exhibit lower expression of inhibitory immune checkpoint receptors than CD8^+^ CAR-T cells [69,70]. One interesting strategy to improve the effectiveness of CAR-T treatments is to alter T cell polarization towards helper T cells. GATA3 plays a crucial role in Th2 T cell function and has been found to positively correlate with the long-term persistence of CAR-T cells in humans [9]. A composite score of activity within the IL-6/STAT3 pathway, a crucial path in Th2 differentiation among CAR-T cells, has been linked to favorable clinical responses [8].

Researchers utilized CAR-T cells under conditions that facilitated Th9 polarization. Compared with traditional CAR-T cells after extended *in vitro* culture, these Th9 T cells exhibited elevated levels of IL9, TNFA, and IL-2, reduced levels of IFNG, and a less differentiated phenotype. In a humanized mouse model, these Th9-polarized CAR-T cells also demonstrated superior efficacy in comparison to standard CAR-T cells [71].

### Innate-like T lymphocytes (γδ T cells and NKT cells)

In recent years, there has been a growing interest in the potential of innate-like T cells, such as γδ T cells and NKT cells, in CAR-T cell-based therapies. These cells have been shown to possess unique functional advantages, primarily due to their expression of NK receptors, which not only enhance their cytotoxic abilities but also provide an added layer of specificity in targeting tumors [72]. Future strategies should focus on refining their functional advantages and addressing challenges associated with tumor heterogeneity to enhance efficacy.

Studies have demonstrated that innate-like T cells exhibit profound antitumor immunity and possess the ability to modify the tumor microenvironment. For instance, in mouse models, these cells have been shown to exert cross-priming effects, influencing the activation and function of other immune cells within the tumor microenvironment [73]. This phenomenon, along with their ability to target tumor cells independently of CAR-targeting antigens, brings hope for overcoming the challenges associated with tumor heterogeneity and antigen escape.

Furthermore, a recent study has shed light on the regulatory effects of IL-23 receptor expression on γδ T cells, indicating that these cells not only possess direct cytotoxic effects but also modulate adaptive immune responses, thus having potential implications in addressing challenges associated with autoimmune diseases [74]. Moreover, NKT cells play a crucial role in the antitumor immune response, exerting CD1d-dependent tumor cytotoxicity and interacting with various immune cells within the tumor microenvironment [75,76]. However, the effectiveness of endogenous NKTs or unmodified NKTs transferred adoptively has been limited, and there is potential for improvement through methods redirecting NKTs using synthetic receptors specifically designed for targeting tumors [72]. These reports emphasize the important role of innate-like T lymphocytes in immune regulation and suggest their potential as targets for CAR-T based immunotherapeutic interventions.

### Cord blood-derived hematopoietic stem cells

A key challenge of autologous CAR-T cell therapy is the difference in the quality of blood-derivedT cells among patients [8]. Similarly, when expanded *in vitro* for therapeutic purposes, non-genetically modified tumor-infiltrating lymphocytes are often hypofunctional and differentiated [77]. Allogeneic, “off-the-shelf” cell therapies from healthy donors are now being developed to address this issue [78]. A key obstacle associated with“off-the-shelf” CAR-T cell therapies produced from mature T cells derived from blood is the necessity for substantial *ex vivo* expansion to generate large cell batches. This process is a limiting step in the proliferative capacity and functionality of these cells in patients. Novel manufacturing techniques have been implemented to optimize the expansion and tumor-killing capabilities of CAR-T and CAR-NKT cells *in vivo* [79,80]. For this purpose, employing CD34^+^ hematopoietic stem cells derived from umbilical cord blood and transduced with a non-alloreactive iNKT TCR as the initial material offers several distinct advantages [79]. It eliminates the requirement to suppress the expression of the native TCR in an allogeneic setting. Additionally, it enables substantial cell expansion during the manufacturing process without inducing terminal differentiation of the final product, as most of the expansion phase occurs before the development of mature, functional T cells.

## Conclusion

The investigation of CAR-T cell therapy has opened a new horizon for the treatment of patients with hematologic malignancies and solid tumors. The T-cell-enhancing approaches represent an important avenue for achieving sustained clinical benefits. However, several challenges exist, and the aforementioned technological advancements have the potential to address these challenges that are associated with premature exhaustion and insufficient antitumor efficacy in cell therapies. Ongoing clinical studies aiming to enhance CAR-T cells’functionality explore the tumor microenvironment, including tumor-associated hypoxia, acidity, altered lipid metabolism, and metabolomics. The focus of further research should be on optimizing T-cell-enhancing approaches and developing highly effective strategies that can potentially cure patients with hematological diseases and, eventually, those with solid tumors, translating scientific advancements into transformative, improved CAR-T cell therapies.
